# High levels of soluble CD40 ligand and matrix metalloproteinase-9 in serum are associated with favorable clinical evolution in human visceral leishmaniasis

**DOI:** 10.1186/1471-2334-13-331

**Published:** 2013-07-19

**Authors:** Fabrícia Alvisi de Oliveira, Carla Vanessa Oliveira Silva, Nayra Prata Damascena, Rodrigo Oliveira Passos, Malcolm S Duthie, Jeffrey A Guderian, Ajay Bhatia, Tatiana Rodrigues de Moura, Steven G Reed, Roque Pacheco de Almeida, Amélia Ribeiro de Jesus

**Affiliations:** 1 Molecular Biology Laboratory, Hospital Universitário – Universidade Federal de Sergipe, Rua Claudio Batista s/n, Bairro Sanatório, Aracaju, Sergipe 49060-10, Brazil; 2 IDRI- Infectious Disease Research Institute, Seattle, WA 98102, USA; 3 Instituto de Investigação em Imunologia (iii) – Institutos Nacionais de Ciência e Tecnologia (INCT), CNPq, São Paulo, Brazil

**Keywords:** Visceral leishmaniasis, Soluble CD40 ligand, Matrix metalloproteinase-9, Biomarkers, Clinical outcome

## Abstract

**Background:**

Soluble CD40 ligand (sCD40L) and matrix metalloproteinase 9 (MMP-9) are inflammation markers and have been poorly described in infectious disease. In this prospective study, we describe the sera kinetics of these two molecules in the course of treatment follow up in human visceral leishmaniasis (VL).

**Methods:**

Sera from VL patients were collected before and during follow up of regular Antimony treatment. sCD40L and MMP-9 were measured by Luminex assay. Paired analysis by Wilcoxon signed test was used for comparison of values of the same subjects before and after initiation of treatment. Correlations between clinical data and parasite load with the serum levels of sCD40L and MMP-9 were performed by Spearman test. Tests were considered statistically significant if the probability of a type I error was less than 5% (*p*-value < 0.05).

**Results:**

While sCD40L and MMP-9 were not observed in sera from non endemic controls which are at low risk of *Leishmania chagasi* infection, elevated levels were observed in sera from VL patients, and an increase in sCD40L and MMP-9 levels were detectable during the follow-up of VL patients undergoing antimony treatment. sCD40L levels were also high in individuals living in endemic settings at high risk of infection (endemic controls). Additionally, negative correlations were found between spleen sizes and MMP-9 before treatment and sCD40L at day 15 of treatment. Negative correlations were also found between parasite load with both sCD40L and MMP-9.

**Conclusion:**

Serum sCD40L and MMP-9 are identified as new and simple biomarkers in two situations: (i) monitoring the success of therapy and (ii) predicting favorable clinical outcome of human VL.

## Background

Visceral leishmaniasis (VL) is a disease caused by systemic infection with protozoa of the genus *Leishmania,* transmitted to mammalian hosts by phlebotomine sandflies*.* Human VL is characterized by fever, hepatosplenomegaly, anemia, leukopenia, and severe weight loss and is fatal if not treated. VL is associated with a marked impairment of leishmania-specific Th1 response, as evaluated by *in vitro* leishmania antigen stimulation of peripheral blood mononuclear cells (PBMC) [1]. However, high levels of proinflammatory cytokines, including IFN-γ, have been detected in serum and bone marrow of VL patients [2-4]. IL-12 and IFN-γ have been associated with a protective immunity in individuals living in an endemic area [5]. These cytokines activate macrophages to release several microbicidal agents to kill the parasites [6]. High amounts of IL-10 are also detected in sera and bone marrow of VL patients, and this cytokine is well known to have an immunosuppressive effect on the leishmania-specific response in VL patients [7-9] and directly correlates to parasite load [10]. The production of IL-10, IL-12 and IFN-γ is dependent on cellular associated costimulatory molecules such as CD40 and CD40L [11].

CD40 was identified on B cells, monocytes, dendritic cells, endothelial and epithelial cells [12]. Cellular CD40L, a member of the TNF family, is primarily expressed on activated CD4+ T cells and on a small proportion of CD8+ T cells and platelets [12]. The interaction of CD40 and CD40L is important for activating antigen presenting cells, T cells and macrophages [12]. CD40-CD40L signaling has an important role during various parasite infections (reviewed in [13]). While strong CD40-CD40L signaling induces IL-12 and IFN-γ production and drives T cells to a Th1 phenotype, weak signaling induces IL-10 and TGF-β, allowing T cells to differentiate to a Th2 or T regulatory phenotype (reviewed in [14]). In the context of leishmaniasis, it is known that *L. major* amastigotes deviate the cellular CD40 signaling pathway by inducing ERK 1/2 and IL-10 production, which inhibits the p38MAPK/IL-12 pathway [15].

CD40-CD40L signaling is also important for antibody isotype switching, and germinal center formation, and patients with congenital X-linked hyper IgM syndrome (X-HIM), caused by the lack of functional CD40L, present an increased incidence of infections with opportunistic pathogens (reviewed in [13]). Peripheral blood mononuclear cells (PBMC) from X-HIM patients also secrete markedly lower amounts of IFN-γ and IL-12 in response to *T. gondii*[16]. CD40L knockout (^-^/^-^) mice are more susceptible to *L. major* infection [17], while mice treated with molecules that bind CD40, thereby mimicking the effects of soluble CD40L, exhibit a decreased severity of experimental *L. major* or *T. cruzi* infection [17,18]. The CD40-CD40L signaling pathway also is involved in matrix metalloproteinases (MMPs) expression, including MMP-9 [19,20].

CD40L may be expressed as an heteromultimeric complex [21]. After cleavage of the transmembrane protein by naturally occurring MMPs, the soluble form present in the plasma (sCD40L) still binds to CD40 and delivers biological signals [22]. This molecule binds cell surface CD40 and induces a strong activation of antigen presenting cells (APC) independent of T cell help, resulting in IL-12 and IFN-γ production [23].

MMPs are produced by different cell types, including macrophages, lymphocytes, endothelial cells, and are associated with remodeling and modulation of inflammation [24]. In tuberculosis, MMP-9 is required for macrophages recruitment and tissue remodeling for the formation of tight well-organized granulomas [25]. MMP-9 also contributes to the myocarditis induced by *T. cruzi,* by favoring the infiltration of immune cells [26].

sCD40L and MMP-9 have been described in cardiovascular disease as mediators of inflammation in arterial coronary disease, and are considered markers of poor prognosis [27,28]. In infectious diseases, increased sCD40L levels are detected in the serum of HIV-1 and Sepsis, and are associated with poor prognosis [29,30]. In leishmaniasis, MMP-9, along with other leishmanicidal products are released by infected macrophages in vitro [31]. Furthermore, in canine visceral leishmaniasis high levels of serum MMP-9 is associated with multi-systemic inflammatory lesions [32]. However, the role of MMP-9 and sCD40L has not been explored in human visceral leishmaniasis. In this novel study, we describe the serum levels of sCD40L and MMP-9 in VL patients in different stages of disease and in post treatment clinical evolution. We also compare their levels with individuals without disease but living in endemic areas and at the same household of the VL cases.

## Methods

### Study design and ethics issues

This was a prospective study performed in Hospital Universitário – Universidade Federal de Sergipe – Brazil, approved by the Ethics Committee of University Hospital from Universidade Federal de Sergipe. All individuals, or their legal guardians, signed an informed consent form. Pregnant women, patients receiving immunosuppressive treatments and patients with comorbidities (including HIV infection and malignancy) were excluded.

### Study population and follow-up

Initially, 45 patients (mean ± SD 15 ± 15 years; 25 males and 20 females) with confirmed VL were selected for this study. The diagnostic criteria used for inclusion were identification of *Leishmania* on bone marrow aspirates by direct exam and culture in NNN media (Sigma-Aldrich, St Louis, MO), and rK39 serological test (Kalazar Detect® Rapid Test; InBios International Inc., Seattle, WA). Patients were examined using a standard protocol containing the following information: identification, clinical complaints, physical exam, and the results of laboratory tests performed (distinct from data used in this study). After diagnosis, patients received regular Antimony treatment (20 mg Sb^v^/Kg for 20 days). Liver and spleen sizes were evaluated by two observers by palpation and reported as the distance to the rib border in cm at diagnosis and every five day during the treatment until the end of therapy, and later, every 3 months until one year post treatment.

Several groups were included to serve as controls. A group of individuals (either household contacts or relatives) living in the same area as the patients but with no signs of clinical disease were recruited (endemic controls) (n = 37). Healthy subjects, United States Citzens, thus not exposed to the infection (non endemic controls) (n = 24) were also included. Sera of chronic Chagas disease patients (n = 29) were obtained from a reference laboratory for diagnosis of public health diseases. Clinical data were not available regarding serum from patients with Chagas disease.

### Laboratorial data

Blood samples without anticoagulant were collected for all study participants, for VL patients, blood was collected before specific leishmania therapy, day 0 (D0) (n = 45) and at several times after the initiation of treatment (D15, n = 42; D30, n = 35; D45, n = 36; D60, n = 30; and D180, n = 11). Sera were collected and stored at -70°C until sCD40L, and MMP-9 concentrations were determined by a Luminex assay, performed according to the manufacturer’s instructions (Millipore, Massachusetts, USA). The levels of sCD40L were measured in all sera samples collected from VL patients, while MMP-9 concentrations were determined in D0, n = 37; D15, n = 30; D30, n = 36; D45, n = 29; D60, n = 29; and D180, n = 6. The measurement of transaminases (ALT and AST) by a chemistry system (Vitros® 5.1 FS - Ortho-Clinical Diagnostics - Johnson & Johnson, New York, USA) was adopted as a hepatic damage marker before treatment in VL patients and was also tested in endemic controls (n = 30; n = 29, respectively). The few missing values for laboratorial data were due to samples limitations.

### Parasite load

Evaluation of parasite load was carried out in blood samples of VL patients before treatment D0 (n = 15), at D30 (n = 13) and D60 (n = 13) of treatment. The blood samples were collected in tubes containing Paxgene (PreAnalytiX GmbH, Hombrechtikon), according to manufacture’s instructions. Nucleic acid extraction was performed by using a QIAamp DNA, Blood Mini Kit (Qiagen, Valencia, CA). The Real-time PCR based on TaqMan Probe was applied for accurate quantification of Leishmania in blood samples of VL patients, described previously[33]. Specific primers based on conserved region of Leishmania 18S ribosomal DNA gene were disigned by using ABI FileBuilder 3.1 and consisted of; forward 5′- CCGTTTCGGCTTTTGTTGGTTTTAA; GCGATGGGAAAGCACTTGTC-3′; and reporter: CAGCTCCATAATCTCC (Applied Biosystems Inc., Foster City, CA). Standards and a no template control were included in each run. Patient samples were first examined in duplicate, then, samples that did not amplify, or had very late amplification, were re-examined in triplicate.

### Statistical analysis

Kolmogorov-Smirnov normality test was applied. Data were analyzed for different groups (non-paired analysis) by a nonparametric Kruskall-Wallis with post Dunn’s multiple comparison test. Student T test for parametric or Mann–Whitney for the non-parametric samples was used for two groups comparisons. Paired analysis by Wilcoxon signed test was used for comparison of values of the same subjects before and after initiation of treatment. Correlations between clinical data and parasite load with the serum levels of sCD40L and MMP-9 were performed by Spearman test. All tests were carried out using Graph Pad Prism, version 4.0, 2005. Tests were considered statistically significant if the probability of a type I error was less than 5% (*p*-value < 0.05).

## Results

### Demographical and clinical data of VL patients

Demographic and clinical data of VL patients before treatment are presented in Table 1. The mean ± SD of age was 15 ± 15 years. Fifty five percent of patients were males. The mean ± SD of spleen size was 9.1 ± 4.08 under the left costal rib. All patients had diagnosis of VL confirmed by rK39 serology, 17 of the patients had bone marrow culture for leishmania performed and were confirmed as positive, and parasite load was assessed in 15.

**Table 1:** Demographic and clinical characteristics of VL patients

**Variables (pre-treatment)**	**VL patients (n = 45)**
Age (mean ± SD)	15 ± 15 years
Males n (%)	25 (55.5%)
Spleen size (mean ± SD)	9.1 ± 4.08
White blood cell count	3035 ± 1351
Neutrophils	1041 ± 745.2
Platelets	181, 21 ± 63,844
Hb	8.475 ± 1.264
Confirmatory of VL	
r K39 (n = 45)	100%
L eishmania culture (n = 17)	100%
Parasite load (n = 15)	100%
AST (mean ± SD)	119.6 ± 188.4U/ml
ALT (mean ± SD)	79.20 ± 103.4U/ml

*Hb* hemoglobine.

*VL* Visceral leishmaniasis.

rK39 serological test specific for Kalazar.

*AST* Aspartate aminotransferase.

*ALT* Alanine aminotransferase.

### Higher levels of sCD40L and MMP-9 in VL patients

**Figure 1:**
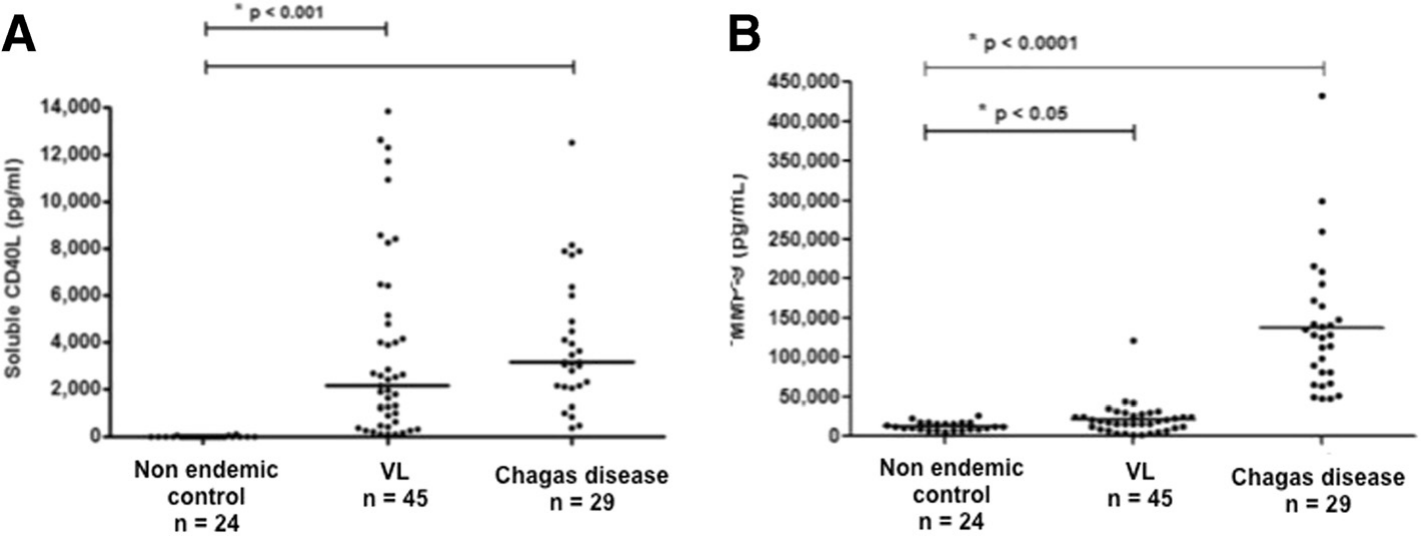
**Serum levels of sCD40L and MMP-9 in VL patients, Chagas disease patients and non endemic controls. (A)** Compares sCD40L levels in non endemic controls, VL patients and Chagas disease patients as measured by Luminex assay (*p* <0.001). **(B)** Compares MMP-9 in non endemic controls and VL patients (*p* < 0.05), and Chagas disease patients (*p* < 0.0001) *Mann Whitney test.

Given that VL and Chagas disease are inflammatory diseases that commonly present with organomegaly, it was not surprising that all patients had detectable levels of circulating sCD40L (Figure 1A). In contrast, among 24 non endemic controls, only 5 presented with detectable serum levels of sCD40L (Figure 1A). It was observed that both Chagas disease and VL patients presented high serum levels of sCD40L (mean ± SD; 3,935 ± 2,804 pg/ml and 3,579 ± 3,878 pg/ml, respectively) and MMP-9 (mean ± SD; 138,151 ± 85,242 pg/ml and 21,815 ± 20,164 pg/ml, respectively); levels significantly higher than those of the non endemic controls (mean ± SD of sCD40L was 8.8 ± 20.26 pg/ml; and of MMP-9 was 13,393 ± 5,363 pg/ml) (*p* < 0.05) (Figure 1). Thus, both sCD40L and MMP-9 levels were evaluated in sera of Chagas disease and VL patients compared with those found in non endemic controls.

### Protective role of sCD40L and MMP-9

**Figure 2:**
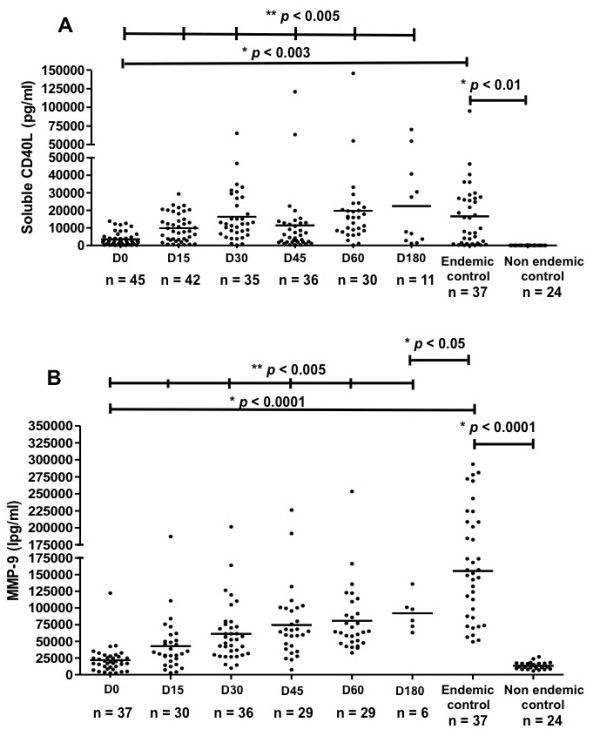
**Serum levels of sCD40L and MMP-9 in follow up of VL treatment and of endemic control subjects. (A)** Compares sera levels of sCD40L and **(B)** MMP-9 in VL patients before (D0) and during treatment follow-up (D15, D30, D45, D60, D180) as measured by Luminex assay. *Mann–Whitney test and ** Wilcoxon signed rank test.

Endemic control subjects had serum sCD40L (mean ± SD; 16,602 ± 19,066 pg/ml) and MMP-9 (mean ± SD; 155,426 ± 72,757 pg/ml) levels higher than non endemic controls (*p* < 0.001). Interestingly, levels of these analytes in endemic controls were also higher than those in VL patients (*p* < 0.005) (Figure 2). These data suggest a protective role of these molecules. This hypothesis is supported by the observation that, when sCD40L and MMP-9 levels were analyzed throughout VL treatment, a significant increase was detected throughout treatment (*p* < 0.005) (Figure 2). At 15, 30, 45, 60 and 180 days after treatment initiation, the levels of sCD40L in VL patients were similar to those of the endemic control subjects (Figure 2A). MMP-9 levels also increased progressively from day 15 to 180 after treatment initiation, but still remained lower than the endemic controls (*p* < 0.05) (Figure 2B).

To further evaluate a potential protective role of sCD40L and MMP-9, we compared the sera levels of these molecules in VL patients with spleen and liver sizes.

**Figure 3:**
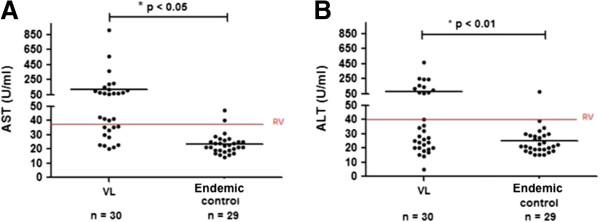
**Serum levels of aspartate aminotransferase (AST) and alanine aminotransferase (ALT) in VL patients and endemic controls. (A)** Compares the levels of AST (*p* < 0.05) and **(B)** ALT (*p* < 0.01) between VL patients and the endemic controls. * Mann Whitney test. RV; reference value.

No significant correlation was found between serum levels of sCD40L and MMP-9 and liver size (data not shown). In order to evaluate if MMP-9 is involved in hepatocyte damage, the sera levels of transaminases (ALT and AST) were correlated with the sera levels of sCD40 and MMP-9 from VL patients before treatment and from endemic controls. Although VL patients showed levels of ALT (79.20 ± 103.4 U/ml) and AST (119.6 ± 188.4 U/ml), higher than those of the endemic controls (24.93 ± 11.45 U/ml; 23.38 ± 6.99 U/ml, ALT and AST, respectively) (Figure 3). No significant correlations were observed between the levels of these enzymes and the sCD40L or MMP-9 levels. In fact, despite having higher levels of MMP-9 in their sera, the endemic controls did not present altered transaminases.

**Figure 4:**
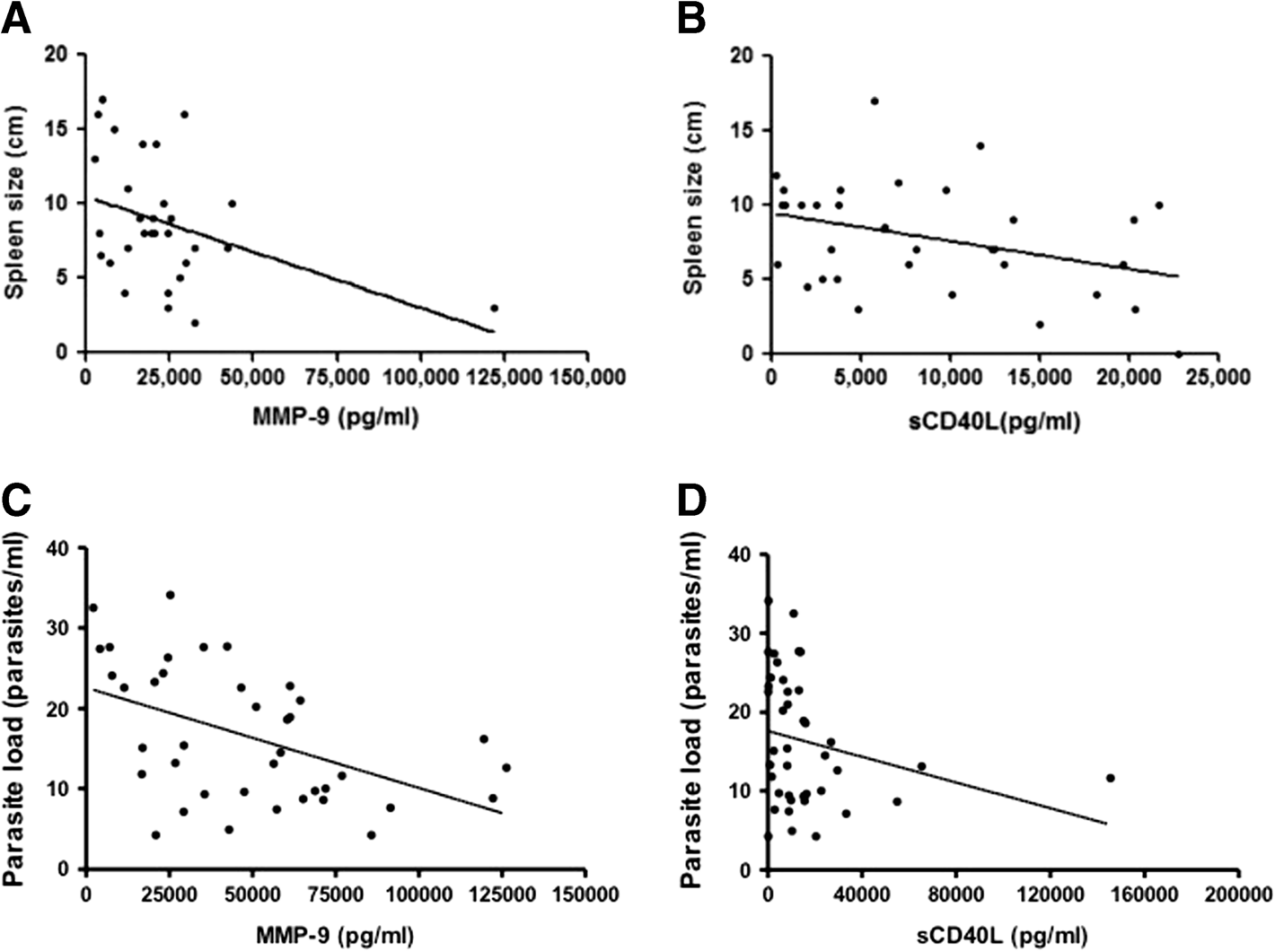
**Correlation between sCD40L and MMP-9 levels with spleen size or parasite load during VL treatment. (A)** Correlation between MMP-9 and spleen size before treatment (n = 30; r = −0.38; *p* < 0.05). **(B)** Correlation between sCD40L and spleen size at D15 of treatment (n = 33; r = −0.36; *p* < 0.05). **(C)** Correlation between sCD40L with parasite load at D0, D30, D60 (n = 41; r = −0.34; *p* < 0.05). **(D)** MMP-9 with parasite load at D0, D30, D60 (n = 39; r = −0.50; *p* < 0.001). Spearman correlation test.

A negative correlation between spleen size and serum levels of MMP-9 was observed before treatment (n = 30; r = −0.38; *p* < 0.05). Similarly, sCD40L levels and spleen size correlated at D15 of treatment (n = 33; r = −0.36; *p* < 0.05) (Figure 4A and B). Negative correlations between parasite load and levels of MMP-9 (n = 39; r = −0.50; *p* < 0.001) and sCD40L (n = 41; r = −0.34; *p* < 0.05) were also observed (Figure 4C and D).

## Discussion

sCD40L and MMP-9 are inflammation markers extensively studied in cardiac disease [27,34,35] but there is no previous data regarding the soluble CD40L (sCD40L) during parasitic infections in humans. In this novel study, we described the presence of these two molecules circulating during parasitic infections. Our results demonstrate that sCD40L and MMP-9 are increased in both Chagas disease and VL. The increasing levels in VL patients during treatment follow-up strongly suggest that theses molecules are markers of favorable clinical evolution. Although leishmania-specific delayed-type hypersensitivity skin reactions (DTH) was not performed in endemic control subjects in the present study, asymptomatic infection (DTH positive) is present in 40% of subjects in endemic areas in Brazil and is frequently associated with the presence of a classical VL case in the family or neighborhood [36,37]. In addition, non endemic controls present lower serum levels of sCD40L and MMP-9. Taken together, these data reinforce our hypothesis that high levels of these molecules in endemic controls may have a protective role in clinical evolution from leishmania infection to VL.

Previous animal studies have indicated the importance of the CD40-CD40L signaling pathway for protection against a variety of parasites (reviewed in [13]). CD40 stimulation induces anti-microbial activity against *T. cruzi,* mediated by the production of nitric oxide (NO) and of free radicals, which require IL-12 and IFN-γ production. Mice infected with *T. cruzi* and treated with CD40L exhibit a decrease in parasitemia and in mortality that are accompanied by prevention of the immunosuppression that typically follows *T. cruzi* infection [18].

In leishmania infection, the role of CD40L is clearly demonstrated. Knockout mice have an increased susceptibility to *L. donovani* infection [38]. CD40-CD40L signaling not only regulates immunity but also influences the outcome and response to pentavalent antimony (Sb) treatment, the conventional chemotherapy for VL [38]. CD40L is necessary to generation of IL-12 and INF-γ [11]. It is assumed that IL-12 drives the Th1 cell-associated mechanism and induces IFN-γ, both cytokines guide T cells and blood monocytes into granulomas at parasitized tissue focus and IFN- γ stimulates effector monocytes and macrophages to kill intracellular parasites (reviewed in [39]). Response to Sb therapy requires an intact type Th1 (INF-γ and IL-12 secretion) which depends on CD40-CD40L interaction [11,38]. Moreover, CD40-CD40L simultaneously with TNF-α is a potent inducer of nitric oxide production, which plays a main role in antimicrobial activity[40]. Previous studies suggest that sCD40L is a marker of disease and poor prognosis of various infections, [29,30,41,42]. In sepsis patients, sCD40L higher than 3.5 ng/ml present a worse survival curve, as compared to the patients with lower than this value (Odds ratio of 2.35 for mortality)[29]. Increased sCD40L levels are detected in the serum of HIV infected subjects and cerebrospinal fluid of patients with AIDS dementia [30,41], and is described to inhibit plasmocytoid dendritic cell-derived IFN-α production [43]. The levels of sCD40L also are higher in untreated than in anti-retrovirus treated HIV patients [44]. Our data in VL patients is the first suggesting a protective role of sCD40L. It is possible that in parasitic infections, where the parasites down modulate the protective immune response, the presence of sCD40L is important to restore this response. In fact, a previous study indicates that *L. major* amastigotes modulate the signaling pathway downstream of membrane CD40 engagement by inducing ERK 1/2 and IL-10 production**,** which inhibits the p38MAPK/IL12 pathway [27]. As sCD40L has been described to provide a strong signal to APC [23], it might be effective at restoring the IL-12 production and thereby have a protective role in VL clinical outcome.

During the course of VL infection there is extensive parasite multiplication that results in a high parasite burden in the spleen and liver. The enlargement of the spleen and liver is a cardinal feature of human VL and the return of these organs to their normal impalpable state has long been used to evaluate cure. The reductions of spleen or liver after the initiation of treatment (data not shown) are observed at D15 of treatment, and at this time point, the reduction of spleen size correlates with serum level of sCD40L.

Our data also indicate elevated sCD40L in Chagas disease patients and it is possible that sCD40L levels may be related to the control of parasitemia seen in the chronic stage of disease. Since *Trypanosoma cruzi* infection also produces an intense inflammatory response in diverse tissues including the heart [45], the sCD40L could be associated with chronic inflammation. No clinical data was available for these patients, however, further studies are required to associate these data with clinical outcome.

The CD40-CD40L signaling pathway also is involved in matrix metalloproteinases (MMPs) expression, including MMP-9 [19,20]. Our data also shows increased levels of MMP-9, following the same profile of sCD40L. Thus, the sCD40L, biologically active, may be contributing to increase the sera levels of MMP-9, previously mentioned. High levels of serum matrix metalloproteinases, MMP-2 and MMP-9, were observed in dogs with natural VL, and the authors suggest that these enzymes play a role in multi-systemic inflammatory lesions found in VL [32]. However, in our study we observed that, at D0 of treatment, patients with the highest levels of MMP-9, in their sera, had smaller spleens sizes than patients with lower MMP-9 levels. MMP-9 has an essential function in matrix compounds degradation during the transmigration of host defense cells and during macrophages migration [46,47]. On the other hand, MMP-9 along with others MMPs is involved in regulation of the inflammatory response in several circumstances, including the direct cleavage of immune system proteins [42].

Alternatively, sCD40L is exposed to cleavage by the action of MMPs, liberating soluble CD40L [22]. Thus, in VL, the high levels of MMP-9 can play a role in the cleavage of sCD40L from the cell membrane, promoting the increase of CD40L in its soluble form. In carcinoma cells, sCD40L induced cytotoxicity and is enhanced by inhibition of metalloproteinase cleavage [48]. In this context, it is difficult to know which of the molecule initiate these events, but they are both involved in a regulatory network and associated with a clear protective response.

Confirming their role as a marker of favorable clinical evolution in VL patients, inverse correlations between the serum sCD40L levels and MMP-9 with parasite load were found. Serum levels of IL-10 are also a good marker of disease severity and directly correlated with parasite load, considering its immunosuppressive role of macrophage microbicidal mechanisms. Although further studies are required to understand both the mechanisms by which the sCD40L and MMP-9 influence disease outcome in human VL, and the timing of the release of these molecules in sera after the infection, sCD40L and MMP-9 represent useful biomarkers with which to predict favorable response to VL therapy. In addition, as there is no marker of protection of clinical evolution from leishmania infection to clinical disease, and in some endemic areas it is difficult to assess clinical data of households contacts, sCD40L and MMP-9 represent important predictive clinical outcome biomarkers.

MMP-9 in macrophage-hepatocyte co-culture supernatants is associated with liver damage, and the release of transaminases AST and ALT [31]. Leishmania infection activates macrophages resulting in the release of several leishmanicidal agents, including MMPs, which in excess, can cause severe tissue/organ damage (reviewed in [49]). In fact, VL patients present evidences of liver damage, demonstrated by increase in AST and ALT. However, no correlations are observed between the MMP-9 and transaminases levels in VL patients. To further support that MMP-9 is not involved in hepatocyte damage, endemic controls present high levels of MMP-9 and no markers of hepatic damage are detected in these subjects (normal levels of AST and ALT). Although, MMP-9 is considered a hallmark of inflammation [42], our data suggest that it is not involved in tissue damage.

## Conclusion

This study identifies serum sCD40L and MMP-9 as new and simple biomarkers in two situations: (i) monitoring the success of therapy and (ii) predicting favorable clinical outcome of human VL.

## Competing interests

The authors declare that they have no competing interests.

## Authors’ contributions

FAO helped to collect the data, built the database, performed the statistical analysis and wrote the paper. CVOS, NPD and ROP conduct the treatment follow up of the VL patients, and collected medical data and the serum samples. MSD, JAG and AB performed the immunoassays and helped to draft the manuscript. TRM helped to collect patients samples and draft the manuscript. SGR participated in study design and financed the immunological tests. RPA participated in study design, supervision of clinical study, and conduct the treatment and follow up of the VL patients. ARJ participated in the supervision of clinical study, provide major help to the first author to perform the statistical analysis and in the manuscript preparation. All authors read and approved the final version of the manuscript.

## Pre-publication history

The pre-publication history for this paper can be accessed here:

http://www.biomedcentral.com/1471-2334/13/331/prepub
